# Lower risk of primary Sjogren’s syndrome in patients with dengue virus infection: a nationwide cohort study in Taiwan

**DOI:** 10.1007/s10067-020-05282-2

**Published:** 2020-07-15

**Authors:** Chi-Ching Chang, Yu-Chun Yen, Cheng-Yi Lee, Chiou-Feng Lin, Chao-Ching Huang, Ching Wen Tsai, Ting-Wu Chuang, Chyi-Huey Bai

**Affiliations:** 1 Division of Allergy, Immunology and Rheumatology, Department of Internal Medicine, School of Medicine, College of Medicine, Taipei Medical University, Taipei, Taiwan; 2 Division of Rheumatology, Immunology and Allergy, Department of Internal Medicine, Taipei Medical University Hospital, Taipei, Taiwan; 3 Research Center of Biostatistics, College of Management, Taipei Medical University, Taipei, Taiwan; 4 Epidemic Intelligence Center, Taiwan Centers for Disease Control, Ministry of Health and Welfare, Taipei, Taiwan; 5 Institute of Health Policy and Management, College of Public Health, National Taiwan University, Taipei, Taiwan; 6 Department of Microbiology and Immunology, School of Medicine, College of Medicine, Taipei Medical University, Taipei, Taiwan; 7 Graduate Institute of Medical Sciences, College of Medicine, Taipei Medical University, Taipei, Taiwan; 8 Department of Pediatrics, School of medicine, College of Medicine, Taipei Medical University, Taipei, Taiwan; 9 Department of Molecular Parasitology and Tropical Diseases, School of Medicine, College of Medicine, Taipei Medical University, Taipei, Taiwan; 10 Department of Public Health, School of Public Health, College of Public Health, Taipei Medical University, 252, Wu-Hsing Street, Taipei, Taiwan

**Keywords:** Autoimmune disease, Cohort study, Dengue virus, Risk

## Abstract

The data concerning the association between dengue viruses (DV) infection and autoimmune diseases (ADs) remain unclear and are scarce. This nationwide population-based cohort study assessed the risk of ADs among patients with DV infection. We analyzed Taiwanese medical data from the Registry of the National Notifiable Disease Reporting System of Taiwan’s Centers for Disease Control between 1998 and 2015 and identified patients with DV infection. From the entire general population data in the National Health Insurance Research Database, we randomly selected a comparison cohort that was individual matching by age, sex, residence, and index date. We analyzed the risk of ADs using a Cox proportional hazards regression model stratified by sex, age, and residence. We enrolled 29,365 patients with DV infection (50.68% men; mean age, 44.13 years) and 117,460 age-, sex-, and residence-matched controls in the present study. The incidence rates of organ-specific ADs were nonsignificantly higher in the DV cohort than in the non-DV control cohort. An approximately 70% lower risk of primary Sjogren syndrome (pSS) was evident in the DV cohort than in the non-DV control cohort with an adjusted hazard ratio of 0.30 (95% confidence interval 0.13–0.67) after adjusting for comorbidities in matched design. By contrast, the other systemic ADs were nonsignificantly lower in the DV cohort than in the non-DV control cohort. This nationwide long-term cohort study demonstrated that patients with DV infection had a lower risk of primary Sjogren syndrome than those without DV infection.

**Table:** 

**Key Points** • *This retrospective, longitudinal cohort observational study shows that patients with DV infection had a lower risk of pSS than those without DV infection.* • *The DV cohort had an approximately 70% lower risk of pSS than the control group, with a multivariate-adjusted HR of 0.30.* • *On the basis of this result, we contended that DV infection has a protective effect that reduces the risk of pSS.*

**Electronic supplementary material:**

The online version of this article (10.1007/s10067-020-05282-2) contains supplementary material, which is available to authorized users.

## Introduction

Dengue viruses (DVs), a group of four serologically distinct but related flaviviruses, are responsible for a major emerging viral disease [1]. This mosquito-borne disease has a considerable negative effect on populations in tropical and subtropical areas of the world in terms of illness, mortality, and economic costs, mainly because of the lack of an approved vaccine or antiviral drugs [2]. Infection with one of the four serotypes of DVs results in symptoms ranging from an acute self-limiting febrile illness, dengue fever, to severe dengue hemorrhagic fever or dengue shock syndrome [3, 4].

When a DV infects a host, it may induce the production of autoantibodies because of the structural antigen similarity between viral proteins and self-antigens [5]. The molecular mimicry between the DV protein and self-antigens can cause antibody cross-reactions that lead to platelet dysfunction, endothelial cell apoptosis, defective coagulation, and macrophage activation [6, 7]. The sequential infection with different DV serotypes may alter the cytokine response of cross-reactive CD4+ T cells, resulting in the production of pro-inflammatory cytokines such interleukin (IL)-2, IL-4, IL-6, IL-8, IL-10, IL-13, IL-18, monocyte chemoattractant protein-1, macrophage migration inhibitory factor, transforming growth factor-β, TNF-α, and IFN-γ [8].

Some evidence suggests that DVs play key roles in the pathogenesis of autoimmune diseases (ADs), and various mechanisms have been invoked to explain these observations, including molecular mimicry and an increase in the immunogenicity of autoantigens caused by inflammation in the target organ [6]. Paradoxically, infectious agents may also play both a causative and protective role in the pathogenesis of ADs [8]. Numerous epidemiologic and experimental studies have clarified and extended hygiene hypothesis support the microorganism had a diverse immunoregulatory effect on ADs [8, 9].

Because of the high prevalence of DV infections in Taiwan, we have debated whether DV infection is associated with the development of ADs. In Taiwan, patients with dengue are placed under the surveillance of the Centers for Disease Control, R.O.C. (Taiwan; known as the Taiwan CDC) which entails being subject to a routine laboratory-based screening and diagnosis system [10]. All hospital-diagnosed cases of dengue must be reported to the Taiwan CDC for confirmation and subsequent surveillance. With respect to whether DVs are important in the pathogenesis of ADs, we used a nationwide population-based dataset of insurance claims to investigate the association of ADs among patients with DV infection.

## Methods

### Data sources

Medical claims data were obtained from the National Health Research Institutes (NHRI). After receiving approval for this study from the NHRI, we used scrambled patient identification numbers to assess the data, including inpatient care claims and the Registry for Beneficiaries. The NHRI maintains and updates the National Health Insurance (NHI) Research Database (NHIRD). The insurance program maintains contracts with 97% of the hospitals and clinics in Taiwan [11]. The accuracy and high validity of diagnoses in the NHIRD have been evaluated [12, 13]. The International Classification of Diseases, Ninth Revision, Clinical Modification (ICD-9-CM) codes were used as the diagnosis codes in the present study. The claims data for all 23,740,000 insured persons were used to establish the study cohorts. The research protocol was approved by the Taipei Medical University-Joint Institutional Review Board (N201602014) and was performed in accordance with the approved guidelines. The need for written informed consent was waived by the Institutional Review Board that approved this study’s protocols. Informed consent from the study patients was not required because the dataset consisted of deidentified secondary data released for research purposes.

### Study cohort

#### Dengue case

Dengue fever is a category 2 notifiable infectious disease in Taiwan and must be reported to the Taiwan CDC by physicians within 24 h of identifying a suspected case. Suspected dengue cases are confirmed by the Taiwan CDC on the basis of the presence of anti-dengue IgM, nucleotide sequence, or viral isolation. Since 2014, nonstructural protein 1 antigen detection has been used as a rapid diagnostic technique in Taiwan. The patient’s travel history is also obtained to clarify whether the case was imported or locally acquired. Only indigenous cases retrieved from the National Notifiable Disease Reporting System of the CDC between 1998 and 2015 were included in the study. The historical medical records (both inpatient and outpatient) of the dengue-confirmed cases can be accessed from the NHIRD. The index date for the DV cohort was identified as the date on which a patient received the first diagnosis of DV infection, as documented in the Registry of the National Notifiable Disease Reporting System. Patients who had been diagnosed with ADs before or within 1 year of DV diagnosis were excluded from this study. The data consolidation and control group selection were processed anonymously in the Department of Statistics, Ministry of Health and Welfare.

### Comparison group

For each patient with a diagnosis of DV infection, a maximum of four comparisons was randomly selected from the NHIRD, a pool of approximately 23.7 million individuals. For the comparison cohort, the index date corresponded to the date on which controls utilized the NHI services. The comparison cohort was age (±2 years), sex (exact), residence (exact), and index date-matched to the DV cohort. Patients diagnosed with an AD before were excluded. The objective of this matching process by propensity score was to guarantee a similar baseline follow-up duration.

### Study endpoints

Each study patient was followed until one of the following outcomes occurred: an AD was diagnosed, the patient was lost to follow-up, the patient died, and the patient withdrew from the NHI system. We identified patients with ADs using ICD-9-CM codes. ADs in this study were categorized into two broad types: systemic and organ-specific ADs [14]. The systemic ADs included Sjogren syndrome (SS; ICD-9-CM code 710.2), psoriasis (ICD-9-CM codes 694.3, 696.0, and 696.1), rheumatoid arthritis (RA; ICD-9-CM code 714.0), systemic lupus erythematosus (SLE; ICD-9-CM code 710.0), scleroderma (ICD-9-CM code 710.1), and polymyositis (PM; ICD-9-CM code 710.4). In Taiwan, patients with systemic ADs (except ankylosing spondylitis ICD-9-CM code 720.0, 720.2, 720.8, 720.9, and psoriasis ICD-9-CM codes 696.0, 696.1, and 694.3) are eligible for a catastrophic illness certificate after receiving the diagnosis from a rheumatology specialist based on their clinical manifestations, laboratory data, and international criteria; the certification requires the precise fulfillment of the related classification criteria [15–24]. The organ-specific ADs included Addison’s disease (ICD-9-CM code 255.4), autoimmune hemolytic anemia (ICD-9-CM code 283.0), diabetes mellitus type 1 (DM; ICD-9-CM code 250.0, 357.2, 366.41, 583.81), Graves’ disease (ICD-9-CM code 242.0), Hashimoto’s thyroiditis (ICD-9-CM code 245.2, 780.01, 244.8, 244.9), Henoch–Schonlein purpura (ICD-9-CM code 287.0), immune thrombocytopenic purpura (ICD-9-CM code 287.3), autoimmune hepatitis (ICD-9-CM code 571.49), myasthenia gravis (ICD-9-CM code 358.0), and inflammatory bowel disease (IBD; ICD-9-CM codes 555 and 556). A person was considered to have a new onset of an organic AD only if the condition occurred in an inpatient setting or was noted in three or more outpatient visits.

In addition, patients with the comorbidities of SLE, RA, scleroderma, PM, DM, a history of head and neck radiation treatment, hepatitis C infection, AIDS, pre-existing lymphoma, sarcoidosis, graft versus host disease, and anticholinergic drug use were excluded to limit our study sample to primary SS (pSS). Therefore, the catastrophic illness patient data are highly accurate and reliable [25, 26].

### Statistical analysis

We compared the frequency or mean of demographic status (age, comorbidity, residence, sex, and periodontitis) between the DV and non-DV cohorts using the chi-squared test or *t* test. The standardized mean differences (SMD) in a matched sample were also shown. The incidence rates of organ-specific and systemic ADs were estimated during the follow-up duration in the DV and non-DV cohorts. The stratified Cox proportional hazards regression model was used to estimate the corresponding hazard ratios (HRs) and 95% confidence intervals (CIs). The matched age, gender, and residence are stratified, and CCI score and periodontitis are adjusted in the Cox regression model. Besides, for the type 1 error problem of multi-outcomes especially under the category of systemic autoimmune diseases, the Hochberg corrected *p* was calculated.

Finally, the cumulative incidences of organ-specific and systemic ADs were estimated using the Kaplan–Meier estimator, known as the product limit estimator, in the DV and non-DV cohorts. SAS (version 9.4, SAS Institute, Cary, NC, USA) was used for all the data analyses, and *p* < 0.05 was considered statistically significant.

## Results

### Baseline characteristics of patients with DV infection and comparison cohorts

A total of 29,365 patients with DV infection were selected from the Registry of Disease Reporting System of the Taiwan CDC and 117,460 control subjects were selected from the NHIRD. The mean follow-up duration in patients with DV infection and without DV infection cohorts was 4.52 ± 4.36 and 4.56 ± 4.32 years, respectively.

**Fig. 1:**
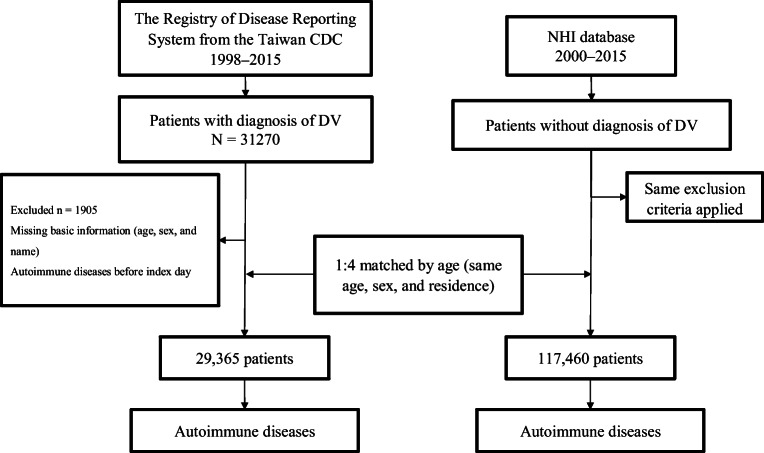
Study design

The flowchart of the study population selection is presented in Fig. 1. The demographic characteristics and baseline comorbidities of the study cohorts are listed in Table 1. The mean ages in the DV and control cohorts were 44.12 ± 19.05 and 44.13 ± 19.05 years, respectively, and the sex ratio of the study population was similar (male 50.68% and female 49.32%). All standardized mean difference in age, gender, and the resident was lower than 0.001. CCI score and periodontitis baseline characteristics were significantly different.

**Table 1:** Baseline characteristics of patients with dengue fever and age, sex, and residence matched to the comparison group

	Comparison group	Dengue group	
	(*n* = 117,460)	(*n* = 29,365)	*p* value
Age			
Mean, SD	44.12, 19.05	44.13, 19.05	0.9984
Age group, *n* (%)			
0–20	15,450 (13.15)	3865 (13.16)	1.0000
20–29	14,817 (12.61)	3711 (12.64)	
30–39	17,731 (15.10)	4426 (15.07)	
40–49	19,759 (16.82)	4936 (16.81)	
50–59	22,977 (19.56)	5741 (19.55)	
60–69	17,339 (14.76)	4352 (14.82)	
70–79	7573 (6.45)	1881 (6.41)	
80	1814 (1.54)	453 (1.54)	
Sex, *n* (%)			0.9897
Male	59,532 (50.68)	14,883 (50.68)	
Female	57,928 (49.32)	14,482 (49.32)	
CCI score, *n* (%)			
Mean, SD	0.62, 1.29	0.78, 1.31	< 0.0001
Min, max	(0, 15)	(0, 14)	
0	77,312 (65.82)	16,306 (55.53)	< 0.0001
1	25,573 (21.77)	8221 (28.00)	
2–3	10,702 (9.11)	3803 (12.95)	
4–6	2833 (2.41)	803 (2.73)	
7–10	810 (0.69)	179 (0.61)	
11	230 (0.20)	53 (0.18)	
Periodontitis	65,118 (55.44)	18,562 (63.21)	< 0.0001
Residence, *n* (%)			1.0000
Kaohsiung	92,696 (78.92)	23,174 (78.92)	
Tainan	13,176 (11.22)	3294 (11.22)	
Pingtung	5364 (4.57)	1341 (4.57)	
New Taipei	1324 (1.13)	331 (1.13)	
Taipei	1272 (1.08)	318 (1.08)	
Taichung	788 (0.67)	197 (0.67)	
Taoyuan	728 (0.62)	182 (0.62)	
Penghu	488 (0.42)	122 (0.42)	
Changhua	344 (0.29)	86 (0.29)	
Yunlin	176 (0.15)	44 (0.15)	
Hsinchu City	172 (0.15)	43 (0.15)	
Nantou	144 (0.12)	36 (0.12)	
Chiayi County	144 (0.12)	36 (0.12)	
Miaoli	136 (0.12)	34 (0.12)	
Taitung	120 (0.10)	30 (0.10)	
Yilan	92 (0.08)	23 (0.08)	
Keelung	88 (0.07)	22 (0.07)	
Hsinchu City	80 (0.07)	20 (0.07)	
Chiayi City	64 (0.05)	16 (0.05)	
Hualien	40 (0.03)	10 (0.03)	
Kinmen, Lienchiang	24 (0.02)	6 (0.02)	

*CCI*, Charlson Comorbidity Index

### Incidence rates and adjusted HRs of ADs in the DV and non-DV cohorts

During the study period, the incidence rate of overall systemic ADs was significantly lower in the DV cohort than in the non-DV cohort (12.60 vs 15.03) with an adjusted HR of 0.81 (95% CI 0.69–0.96) after matching age, gender, residence, and adjusting for comorbidities (Table 2). Furthermore, for various systemic ADs, *n* = 80 and *n* = 7 patients in the non-DV and DV cohorts, respectively, developed pSS. The incidence rate of pSS was lower in the DV cohort than in the non-DV cohort (0.51 vs 1.47), with an adjusted HR of 0.30 (95% CI 0.13–0.67) after matching age, gender, residence, and adjusting for comorbidities. It demonstrated DV patients had 70% less likely to have pSS than control groups significantly (Hochberg *p* < 0.001). By contrast, the other systemic ADs were nonsignificantly lower in the DV cohort than in the non-DV control cohort (Table 2).

**Table 2:** Outcome incidence in patients with DV and comparison group and results of the Cox model regression

	Comparison group		Dengue group			
Outcome	*n* (%)	Incidenceª	*n* (%)	Incidenceª	IRR	Adjusted HR (95% CI)^b^
Systemic autoimmune diseases	813 (0.69)	15.03	172 (0.59)	12.60	0.84	0.81 (0.69–0.96)*
Ankylosing spondylitis	383 (0.33)	7.05	89 (0.30)	6.50	0.92	0.89 (0.70–1.13)
Psoriasis	237 (0.20)	4.36	52 (0.18)	3.79	0.87	0.84 (0.62–1.15)
Inflammatory myopathy	- (0.00)	-	0 (0.00)	0.00		NA
Rheumatoid arthritis	91 (0.08)	1.67	14 (0.05)	1.02	0.61	0.60 (0.34–1.08)
Sjogren’s syndrome	80 (0.07)	1.47	7 (0.02)	0.51	0.35	0.30 (0.13–0.67)** a++
Systemic lupus erythematosus	29 (0.02)	0.53	11 (0.04)	0.80	1.51	1.65 (0.74–3.71)
Systemic sclerosis	11 (0.01)	-	- (0.00)	-		NA
Systemic vasculitis	- (0.00)	-	- (0.01)	-		NA
Organ-specific autoimmune diseases	2212 (1.88)	41.35	597 (2.03)	44.37	1.07	1.03 (0.94–1.13)
Addison’s disease	319 (0.27)	5.87	76 (0.26)	5.55	0.95	0.96 (0.74–1.25)
Autoimmune hemolytic anemia	15 (0.01)	0.28	5 (0.02)	0.36	1.32	1.07 (0.33–3.47)
Diabetes mellitus type 1	894 (0.76)	16.52	236 (0.80)	17.32	1.05	1.00 (0.86–1.16)
Graves’ disease	275 (0.23)	5.06	80 (0.27)	5.84	1.15	1.12 (0.87–1.45)
Hashimoto’s thyroiditis	430 (0.37)	7.92	106 (0.36)	7.75	0.98	0.90 (0.72–1.12)
Henoch–Schonlein purpura	13 (0.01)	0.24	8 (0.03)	0.58	2.44	2.20 (0.83–5.81)
Immune thrombocytopenic purpura	52 (0.04)	0.95	22 (0.07)	1.60	1.68	1.59 (0.94–2.69)
Autoimmune hepatitis	293 (0.25)	5.39	92 (0.31)	6.73	1.25	1.22 (0.95–1.56)
Myasthenia gravis	14 (0.01)	0.26	3 (0.01)	0.22	0.85	0.85 (0.23–3.14)
Inflammatory bowel disease	13 (0.01)	15.03	0 (0.00)	12.60	0.84	NA

ªIncidence per 10,000 person-years

^b^Matched age, gender, residence and adjusted by CCI score, and periodontitis (yes/no)

*For HR, raw *p = 0.03*

**For HR, raw *P* < 0.001. a: Hochberg corrected *p* < 0.001^++^

*CI*, confidence interval; *HR*, hazard ratio; *IRR*, incidence rate ratio; *NA*, not applicable; inflammatory bowel disease (Crohn’s disease or regional enteritis-unspecified, ulcerative colitis), systemic vasculitis (Behçet’s disease, polyarteritis nodosa, Takayasu arteritis, or temporal arteritis)

For organ-specific ADs, the incidence rate of overall organ-specific ADs was nonsignificantly higher in the DV cohort than in the non-DV control cohort (Table 2). Furthermore, the incidence rate of respective organ-specific ADs was also nonsignificantly higher in the DV cohort than in the non-DV control cohort. Detailed information such as model parameters and related standard error, Walt statistics and *p* value were appended in the supplementary table.

### Cumulative incidences of ADs in the DV and non-DV cohorts

**Fig. 2:**
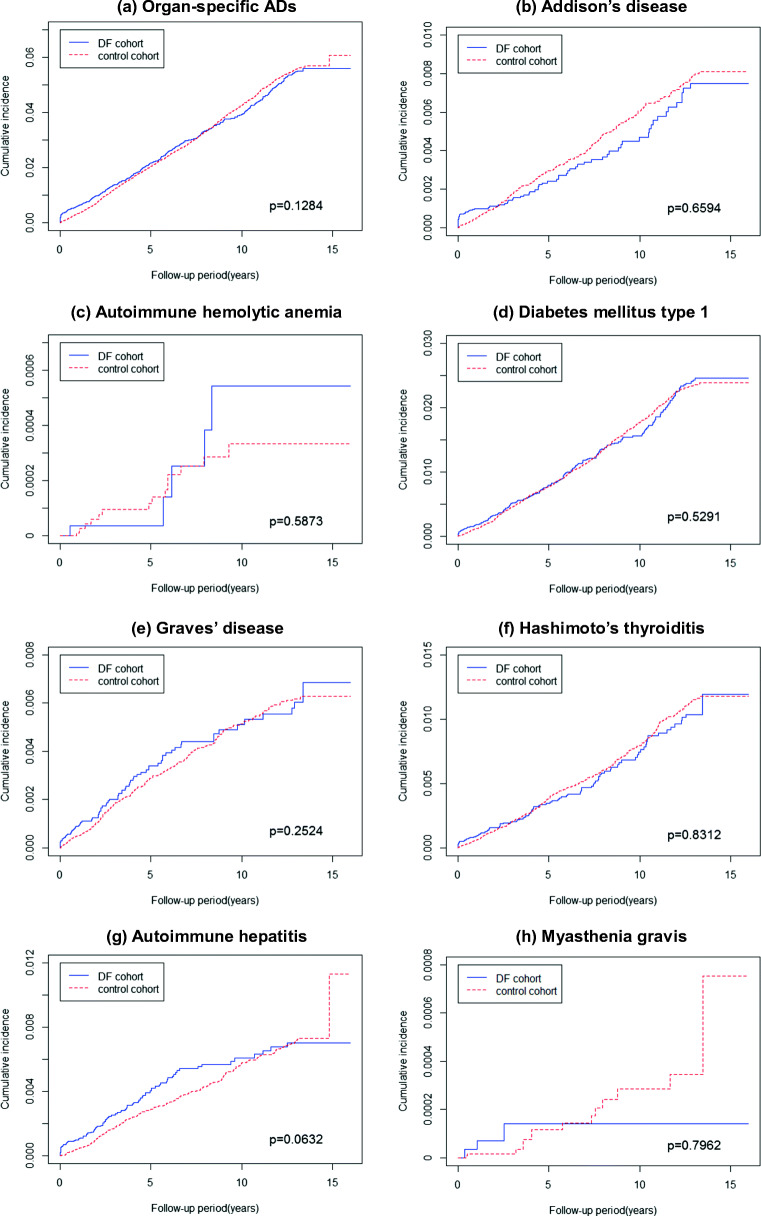
Kaplan–Meier plots of cumulative incidence of organ-specific ADs. **a** Organ-specific ADs. **b** Addison’s disease. **c** Autoimmune hemolytic anemia. **d** Diabetes mellitus type 1. **e** Graves’ disease. **f** Hashimoto’s thyroiditis. **g** Autoimmune hepatitis. **h** Myasthenia gravis

**Fig. 3:**
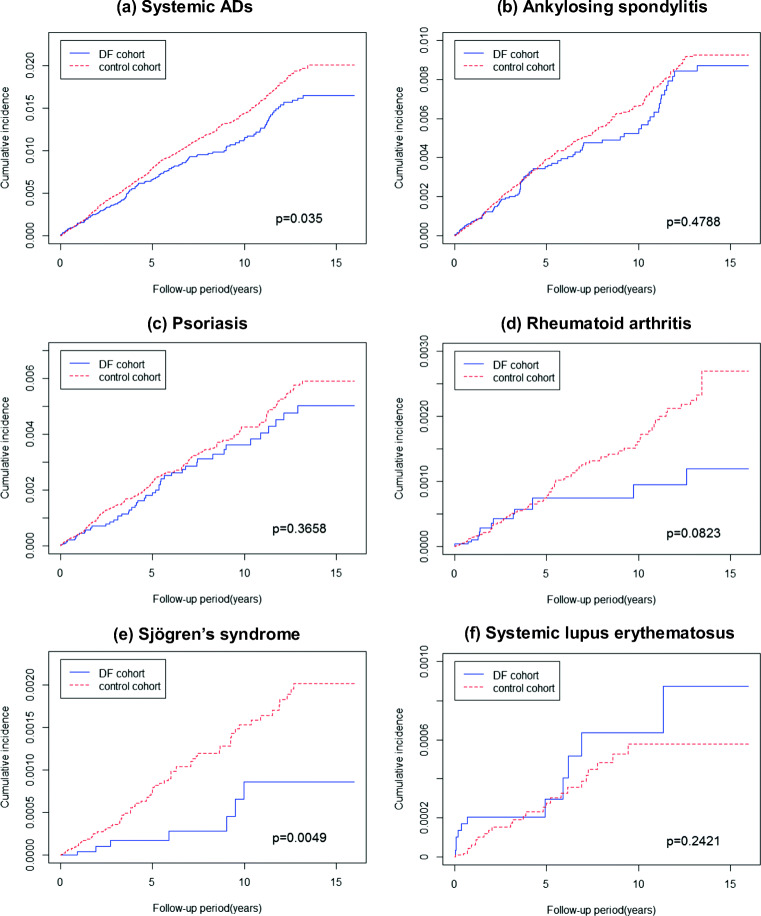
Kaplan–Meier plots of cumulative incidence of systemic ADs. **a** Systemic ADs. **b** Ankylosing spondylitis. **c** Psoriasis. **d** Rheumatoid arthritis. **e** Sjogren’s syndrome. **f** Systemic lupus erythematosus

The comparative cumulative incidence of organ-specific ADs in the DV and non-DV cohorts is presented in Fig. 2. The Kaplan–Meier estimates of overall and respective organ-specific AD-free survival revealed a nonsignificantly higher incidence rate in the DV cohort than in the matched control cohort (Fig. 2a–h). The comparative cumulative incidence of systemic ADs in the DV and non-DV cohorts is presented in Fig. 2. The Kaplan–Meier estimates of overall systemic AD-free survival revealed a significantly lower incidence rate in the DV cohort than in the matched control cohort (log-rank *p* = 0.035; Fig. 3a). Furthermore, the Kaplan–Meier estimates of pSS-free survival revealed a significantly lower incidence rate in the DV cohort than in the matched control cohort (log-rank *p* = 0.0049; Fig. 3e). The Kaplan–Meier estimates of other systemic disease-free survival revealed a nonsignificantly lower incidence rate in the DV cohort than in the matched control cohort (Fig 3 b, c, d, and f).

## Discussion

According to our review of the relevant literature, this is the first nationwide population-based study to evaluate the relationship between DV infection and the risk of ADs. The results of this study demonstrated that patients with DV infection had a lower risk of pSS than those without DV infection. The DV cohort had an approximately 70% lower risk of pSS than the control group, with an adjusted HR of 0.30 after adjusting for age, sex, and comorbidities. On the basis of this result, we contended that DV infection has a protective effect that reduces the risk of pSS.

DV infection can cause abnormal immune responses. Autoimmunity is characterized by autoantibody production and the activation of autoreactive lymphocytes. Other studies have indicated that the onset of the autoimmune response in dengue is a part of the pathogenesis of the disease that can affect various organs and systems [6, 7]. These findings are formulated in a hypothesis concerning the possible role of DVs in the induction and maintenance of autoimmunity in ADs. However, our study determined that DV infection decreases the risk of ADs such as pSS.

Establishing a direct epidemiological association between microbial infections and autoimmune disorders is difficult. Attempts have largely been unsuccessful so far because of multiple predicaments. First, both patients suffering from ADs and healthy individuals undergo multiple infections during their lifetime. Most are cleared by the time of disease diagnosis. Thus, viral infections can be considered “hit and run” events that leave no precise evidence to establish the patient’s history of prior infections. Second, genetic factors such as the MHC haplotype not only are directly responsible for disease susceptibility but also profoundly influence the antiviral immune response. A third factor that adds to the complexity is that infections are less likely to directly initiate autoimmunity but rather accelerate pre-existing autoimmune conditions that then progress to clinical diseases. This implies that multiple sequential events could be necessary to precipitate disease and further complicate attempts to establish firm proof for the involvement of environmental factors. Fourth, the precise timing, location, and magnitude of inflammation and viral strain might all play key roles. Indeed, some studies have demonstrated that the modification of these parameters can change a disease-enhancing viral infection to the one that prevents diabetes [27, 28]. As a last factor, one must recognize that certain infections might protect an individual from an autoimmune response rather than enhance it [29, 30]. Thus, the entire infection history of each patient might determine the overall immune status that results in an AD.

Many experimental systems support the hygiene hypothesis, which postulates that infections caused by viruses and inflammation protect rather than induce/accelerate ADs. For example, injection with coxsackievirus can not only enhance [31] but also prevent diseases in the non-obese diabetic (NOD) mouse [32]. Furthermore, IFN-γ or TNF-α has protective effects in experimental autoimmune encephalomyelitis models or diabetes models when administered late in the disease process [33, 34]. The hygiene hypothesis [35] addresses the relationship between the reduction in the incidence of infectious diseases and the increase in the incidence of allergic diseases and ADs, and the apparent protective effects of infections against immune-mediated diseases have clear clinical implications. A major problem with these correlations is that the infections contributing to protection or susceptibility are ill defined. Moreover, certain infectious agents can trigger allergic diseases or ADs. Although our retrospective study supports the hygiene hypothesis, two lines of research are needed. One should focus on strengthening the epidemiologic evidence, especially through the use of prospective studies. Some allergic diseases and ADs are amenable to prospective epidemiologic investigations because they occur early in life (such as atopic dermatitis, asthma, and type 1 diabetes), thereby reducing the survey time. The second line of research should examine the reduced incidence of selected allergic diseases and ADs by innocuous immunostimulation.

Many animal models have been generated to study the mechanisms of the process that protects an individual from ADs. Mechanistically, several factors may play a role. First, inflammation caused by viruses, bacteria, and especially by parasites such as helminthes can shift the Th1–Th2 balance toward a more immunosuppressive state. In these situations, regulatory T cells might be induced or augmented. Indeed, some studies have discovered evidence for regulatory cells with specificity for pathogens in *Leishmania majo* [36], HSV [37], and Friend retrovirus (murine leukemia virus) infections [38]. Second, inflammation might cause a massive hyperactivation of autoaggressive lymphocytes, which may lead to activation-induced cell death and diminish the systemic load of aggressive T cells. The concept that repeated encounters with strong antigenic stimuli lead to contraction of an immune response is well established in viral infections where the primary response undergoes a major contraction after Ag has been eliminated [39, 40]. These considerations also imply that to enhance autoimmunity, just the right type of stimulus is required. Pushing aggressive T cells too much will result in their rapid death by apoptosis [29], whereas low-level stimuli such as those provided by molecular mimicry might expand dangerous T cell populations by circumventing excessive apoptosis [29]. Third, infection at another location might keep autoaggressive cells from reaching the site of autoimmune destruction. This last possibility may be responsible for the abrogation of T1D in NOD mice after lymphocytic choriomeningitis virus infection, as was initially observed more than a decade ago [41, 42]. Taking all this present evidence together, such animal models form a platform from which possible therapeutic treatments that target the termination or control of the protective process can be evaluated. They can serve as useful tools to understand the mechanisms that could underlie complex human autoimmune disorders and are well suited to establish proof of principle. The aforementioned underlying mechanisms are multiple and complex. They include decreased consumption of homeostatic factors and immunoregulation, involving various regulatory T cell subsets and toll-like receptor stimulation [35]. These mechanisms could originate, to some extent, from changes in microbiota caused by changes in lifestyle, particularly in IBD. Taken together, these data open new therapeutic perspectives for the prevention of ADs.

The pSS is an AD characterized by the activation of minor salivary gland (MSG) epithelial cells and B and T lymphocytic infiltrates. These findings have long encouraged the hypothesis that persistent viral infection of the MSG epithelial cells may drive the autoimmune response; however, the identity of that virus has remained elusive. Over the past decades, an extensive debate has developed concerning the possible role of various viral strains in the induction or maintenance of several ADs, including pSS [43, 44]. Infections caused by coxsackievirus strains B3 and B4 play a role in the initiation of SS [45]. Furthermore, several lines of epidemiological, serological, and experimental evidence implicate retroviral infections, especially HTLV-1, HIVs, HIAP-I, and HRV-5, as triggering factors for the development of SS [46]. However, our study does not support these findings. The relationship between infections and autoimmunity is complex. Current evidence indicates that microbes can initiate, enhance, or conversely, abrogate autoimmunity [47].

The strengths of the present study include large sample size, a large validation cohort, and the long-term ascertainment of concurrent ADs. However, the present study has some limitations. First, although the Bureau of NHI routinely and randomly monitors patient charts to ensure the quality of claims from all medical institutions, the possibility of miscoding or misclassification cannot be completely ruled out. However, such bias would apply to both the DV and control cohorts, and therefore, the present findings are expected to underestimate rather than overestimate the magnitude of the association between DVs and ADs. Second, the relationship between the severity of DVs and ADs could not be analyzed. Additional prospective studies are warranted to confirm whether the severity of DVs increases the risk of ADs. Finally, some important information regarding laboratory or clinical data was not readily available in the administrative database, such as DV infection subtype data. Therefore, the relationship between the various subtypes of DV infection (dengue fever or dengue hemorrhagic fever) and ADs remains unclear. Additional studies are warranted to explore this association.

In conclusion, this nationwide long-term cohort study revealed an association between DV infection and a lower risk of pSS. The protective immune mechanisms of DVs require elucidation. These findings may lead to the development of novel therapeutic strategies for ADs.

## Electronic supplementary material

ESM 1 (DOCX 152 kb)
