# Supplementary material for: Lower risk of primary Sjogren’s syndrome in patients with dengue virus infection: a nationwide cohort study in Taiwan
Source: Clin Rheumatol. 2020 Jul 15;40(2):537–46. doi: 10.1007/s10067-020-05282-2 (PMC7817565; doi:10.1007/s10067-020-05282-2)
Supplement: Supplementary file 1 — (DOCX 152 kb) [file 10067_2020_5282_MOESM1_ESM.docx]

**Supplementary table**

Outcome: Systemic autoimmune diseases

| Parameter | DF | Parameter Estimates | Standard Errors | Chi- Square | Pr>  ChiSq | Hazard Ratio | 95% Hazard Ratio Confidence Limits | |
| --- | --- | --- | --- | --- | --- | --- | --- | --- |
| **Group** |  |  |  |  |  |  |  |  |
| Dengue vs Comparison | 1 | -0.21 | 0.09 | 5.83 | 0.02 | 0.81 | 0.69 | 0.96 |
| **CCI score** |  |  |  |  |  |  |  |  |
| 1 vs 0 | 1 | 0.34 | 0.09 | 13.34 | 0.0003 | 1.40 | 1.17 | 1.68 |
| 2~3 vs 0 | 1 | 0.69 | 0.13 | 29.76 | <.0001 | 1.99 | 1.55 | 2.54 |
| 4~6 vs 0 | 1 | 0.31 | 0.25 | 1.57 | 0.21 | 1.36 | 0.84 | 2.20 |
| 7~10 vs 0 | 1 | 0.96 | 0.45 | 4.49 | 0.03 | 2.60 | 1.07 | 6.30 |
| 11~ vs 0 | 1 | 0.60 | 1.16 | 0.27 | 0.60 | 1.82 | 0.19 | 17.70 |
| **Periodontitis** |  |  |  |  |  |  |  |  |
| Yes vs No | 1 | -0.31 | 0.07 | 16.91 | <.0001 | 0.74 | 0.64 | 0.85 |
| **Age** |  |  |  |  |  |  |  |  |
| 20~29 vs 0~19 | 1 | 0.71 | 1.46 | 0.24 | 0.63 | 2.03 | 0.12 | 35.18 |
| 30~39 vs 0~19 | 1 | 2.70 | 1.87 | 2.09 | 0.15 | 14.94 | 0.38 | 586.11 |
| 40~49 vs 0~19 | 1 | 3.15 | 2.02 | 2.44 | 0.12 | 23.33 | 0.45 | 1211.12 |
| 50~59 vs 0~19 | 1 | 3.64 | 2.12 | 2.94 | 0.09 | 37.99 | 0.60 | 2423.30 |
| 60~69 vs 0~19 | 1 | 2.52 | 2.20 | 1.32 | 0.25 | 12.49 | 0.17 | 923.98 |
| 70~79 vs 0~19 | 1 | 0.79 | 2.35 | 0.11 | 0.74 | 2.21 | 0.02 | 223.56 |
| 80~ vs 0~19 | 0 | 0 | . | . | . | . | . | . |
| **Gender** |  |  |  |  |  |  |  |  |
| F vs M | 0 | 0 | . | . | . | . | . | . |
| **Residence** |  |  |  |  |  |  |  |  |
| Kaohsiung | 0 | 0 | . | . | . | . | . | . |
| Tainan | 0 | 0 | . | . | . | . | . | . |
| Pingtung | 0 | 0 | . | . | . | . | . | . |
| New Taipei | 0 | 0 | . | . | . | . | . | . |
| Taipei | 0 | 0 | . | . | . | . | . | . |
| Taichung | 0 | 0 | . | . | . | . | . | . |
| Taoyuan | 0 | 0 | . | . | . | . | . | . |
| Penghu | 0 | 0 | . | . | . | . | . | . |
| Changhua | 0 | 0 | . | . | . | . | . | . |
| Yunlin | 0 | 0 | . | . | . | . | . | . |
| Hsinchu City | 0 | 0 | . | . | . | . | . | . |
| Nantou | 0 | 0 | . | . | . | . | . | . |
| Chiayi County | 0 | 0 | . | . | . | . | . | . |
| Miaoli | 0 | 0 | . | . | . | . | . | . |
| Taitung | 0 | 0 | . | . | . | . | . | . |
| Yilan | 0 | 0 | . | . | . | . | . | . |
| Keelung | 0 | 0 | . | . | . | . | . | . |
| Hsinchu City | 0 | 0 | . | . | . | . | . | . |
| Chiayi City | 0 | 0 | . | . | . | . | . | . |
| Hualien | 0 | 0 | . | . | . | . | . | . |
| Kinmen, Lienchiang | 0 | 0 | . | . | . | . | . | . |

Outcome: Ankylosing spondylitis

| Parameter | DF | Parameter Estimates | Standard Errors | Chi- Square | Pr>  ChiSq | Hazard Ratio | 95% Hazard Ratio Confidence Limits | |
| --- | --- | --- | --- | --- | --- | --- | --- | --- |
| **Group** |  |  |  |  |  |  |  |  |
| Dengue vs Comparison | 1 | -0.12 | 0.12 | 0.96 | 0.33 | 0.888 | 0.70 | 1.13 |
| **CCI score** |  |  |  |  |  |  |  |  |
| 1 vs 0 | 1 | 0.20 | 0.13 | 2.21 | 0.14 | 1.22 | 0.94 | 1.59 |
| 2~3 vs 0 | 1 | 0.71 | 0.18 | 15.17 | <.0001 | 2.04 | 1.42 | 2.91 |
| 4~6 vs 0 | 1 | -0.01 | 0.35 | 0.00 | 0.98 | 0.99 | 0.50 | 1.98 |
| 7~10 vs 0 | 1 | 0.59 | 0.71 | 0.70 | 0.40 | 1.81 | 0.45 | 7.25 |
| 11~ vs 0 | 1 | 1.92 | 1.42 | 1.83 | 0.18 | 6.82 | 0.42 | 110.45 |
| **Periodontitis** | 1 | -0.36 | 0.11 | 10.94 | 0.0009 | 0.70 | 0.56 | 0.86 |
| Yes vs No |  |  |  |  |  |  |  |  |
| **Age** |  |  |  |  |  |  |  |  |
| 20~29 vs 0~19 | 1 | 13.45 | 764.32 | 0.0003 | 0.99 | 697204.7 | 0 | . |
| 30~39 vs 0~19 | 1 | 27.54 | 888.22 | 0.001 | 0.98 | 9.14E+11 | 0 | . |
| 40~49 vs 0~19 | 1 | 27.41 | 888.22 | 0.001 | 0.98 | 8.02E+11 | 0 | . |
| 50~59 vs 0~19 | 1 | 28.42 | 888.22 | 0.001 | 0.97 | 2.20E+12 | 0 | . |
| 60~69 vs 0~19 | 1 | 26.09 | 888.22 | 0.0009 | 0.98 | 2.15E+11 | 0 | . |
| 70~79 vs 0~19 | 1 | 9.91 | 1754 | 0 | 1.00 | 20126.18 | 0 | . |
| 80~ vs 0~19 | 0 | 0 | . | . | . | . | . | . |
| **Gender** |  |  |  |  |  |  |  |  |
| F vs M | 0 | 0 | . | . | . | . | . | . |
| **Residence** |  |  |  |  |  |  |  |  |
| Kaohsiung | 0 | 0 | . | . | . | . | . | . |
| Tainan | 0 | 0 | . | . | . | . | . | . |
| Pingtung | 0 | 0 | . | . | . | . | . | . |
| New Taipei | 0 | 0 | . | . | . | . | . | . |
| Taipei | 0 | 0 | . | . | . | . | . | . |
| Taichung | 0 | 0 | . | . | . | . | . | . |
| Taoyuan | 0 | 0 | . | . | . | . | . | . |
| Penghu | 0 | 0 | . | . | . | . | . | . |
| Changhua | 0 | 0 | . | . | . | . | . | . |
| Yunlin | 0 | 0 | . | . | . | . | . | . |
| Hsinchu City | 0 | 0 | . | . | . | . | . | . |
| Nantou | 0 | 0 | . | . | . | . | . | . |
| Chiayi County | 0 | 0 | . | . | . | . | . | . |
| Miaoli | 0 | 0 | . | . | . | . | . | . |
| Taitung | 0 | 0 | . | . | . | . | . | . |
| Yilan | 0 | 0 | . | . | . | . | . | . |
| Keelung | 0 | 0 | . | . | . | . | . | . |
| Hsinchu City | 0 | 0 | . | . | . | . | . | . |
| Chiayi City | 0 | 0 | . | . | . | . | . | . |
| Hualien | 0 | 0 | . | . | . | . | . | . |
| Kinmen, Lienchiang | 0 | 0 | . | . | . | . | . | . |

Outcome: Psoriasis

| Parameter | DF | Parameter Estimates | Standard Errors | Chi- Square | Pr>  ChiSq | Hazard Ratio | 95% Hazard Ratio Confidence Limits | |
| --- | --- | --- | --- | --- | --- | --- | --- | --- |
| **Group** |  |  |  |  |  |  |  |  |
| Dengue vs Comparison | 1 | -0.17 | 0.16 | 1.1633 | 0.28 | 0.84 | 0.62 | 1.15 |
| **CCI score** |  |  |  |  |  |  |  |  |
| 1 vs 0 | 1 | 0.42 | 0.17 | 6.23 | 0.01 | 1.53 | 1.10 | 2.13 |
| 2~3 vs 0 | 1 | 0.61 | 0.24 | 6.32 | 0.01 | 1.85 | 1.15 | 2.98 |
| 4~6 vs 0 | 1 | 0.38 | 0.44 | 0.73 | 0.39 | 1.46 | 0.62 | 3.45 |
| 7~10 vs 0 | 1 | 0.77 | 0.87 | 0.79 | 0.38 | 2.17 | 0.39 | 11.99 |
| 11~ vs 0 | 1 | -13.05 | 1466 | 0.00 | 0.99 | 0 | 0 | . |
| **Periodontitis** |  |  |  |  |  |  |  |  |
| Yes vs No | 1 | -0.29 | 0.14 | 4.42 | 0.04 | 0.75 | 0.57 | 0.98 |
| **Age** |  |  |  |  |  |  |  |  |
| 20~29 vs 0~19 | 1 | 11.45 | 653.22 | 0.0003 | 0.99 | 93970.86 | 0 | . |
| 30~39 vs 0~19 | 1 | 11.87 | 653.22 | 0.0003 | 0.99 | 142970.4 | 0 | . |
| 40~49 vs 0~19 | 1 | 13.74 | 653.22 | 0.0004 | 0.98 | 926448 | 0 | . |
| 50~59 vs 0~19 | 1 | 15.46 | 653.22 | 0.0006 | 0.98 | 5191392 | 0 | . |
| 60~69 vs 0~19 | 1 | 14.19 | 653.22 | 0.0005 | 0.98 | 1461506 | 0 | . |
| 70~79 vs 0~19 | 0 | 0 | . | . | . | . | . | . |
| 80~ vs 0~19 | 0 | 0 | . | . | . | . | . | . |
| **Gender** |  |  |  |  |  |  |  |  |
| F vs M | 0 | 0 | . | . | . | . | . | . |
| **Residence** |  |  |  |  |  |  |  |  |
| Kaohsiung | 0 | 0 | . | . | . | . | . | . |
| Tainan | 0 | 0 | . | . | . | . | . | . |
| Pingtung | 0 | 0 | . | . | . | . | . | . |
| New Taipei | 0 | 0 | . | . | . | . | . | . |
| Taipei | 0 | 0 | . | . | . | . | . | . |
| Taichung | 0 | 0 | . | . | . | . | . | . |
| Taoyuan | 0 | 0 | . | . | . | . | . | . |
| Penghu | 0 | 0 | . | . | . | . | . | . |
| Changhua | 0 | 0 | . | . | . | . | . | . |
| Yunlin | 0 | 0 | . | . | . | . | . | . |
| Hsinchu City | 0 | 0 | . | . | . | . | . | . |
| Nantou | 0 | 0 | . | . | . | . | . | . |
| Chiayi County | 0 | 0 | . | . | . | . | . | . |
| Miaoli | 0 | 0 | . | . | . | . | . | . |
| Taitung | 0 | 0 | . | . | . | . | . | . |
| Yilan | 0 | 0 | . | . | . | . | . | . |
| Keelung | 0 | 0 | . | . | . | . | . | . |
| Hsinchu City | 0 | 0 | . | . | . | . | . | . |
| Chiayi City | 0 | 0 | . | . | . | . | . | . |
| Hualien | 0 | 0 | . | . | . | . | . | . |
| Kinmen, Lienchiang | 0 | 0 | . | . | . | . | . | . |

Outcome: Rheumatoid arthritis

| Parameter | DF | Parameter Estimates | Standard Errors | Chi- Square | Pr>  ChiSq | Hazard Ratio | 95% Hazard Ratio Confidence Limits | |
| --- | --- | --- | --- | --- | --- | --- | --- | --- |
| **Group** |  |  |  |  |  |  |  |  |
| Dengue vs Comparison | 1 | -0.50 | 0.30 | 2.92 | 0.09 | 0.60 | 0.34 | 1.08 |
| **CCI score** |  |  |  |  |  |  |  |  |
| 1 vs 0 | 1 | 0.48 | 0.28 | 3.02 | 0.08 | 1.62 | 0.94 | 2.77 |
| 2~3 vs 0 | 1 | 0.80 | 0.37 | 4.73 | 0.03 | 2.23 | 1.08 | 4.60 |
| 4~6 vs 0 | 1 | 1.01 | 1.30 | 0.61 | 0.44 | 2.75 | 0.22 | 35.16 |
| 7~10 vs 0 | 1 | -13.91 | 1903 | 0.0001 | 0.99 | 0 | 0 | . |
| 11~ vs 0 | 0 | 0 | . | . | . | . | . | . |
| **Periodontitis** |  |  |  |  |  |  |  |  |
| Yes vs No | 1 | -0.51 | 0.23 | 4.67 | 0.03 | 0.60 | 0.38 | 0.95 |
| **Age** |  |  |  |  |  |  |  |  |
| 20~29 vs 0~19 | 0 | 0 | . | . | . | . | . | . |
| 30~39 vs 0~19 | 0 | 0 | . | . | . | . | . | . |
| 40~49 vs 0~19 | 1 | 0.63 | 2692 | 0 | 1.00 | 1.88 | 0 | . |
| 50~59 vs 0~19 | 1 | -14.33 | 1828 | 0.0001 | 0.99 | 0 | 0 | . |
| 60~69 vs 0~19 | 1 | -14.69 | 1828 | 0.0001 | 0.99 | 0 | 0 | . |
| 70~79 vs 0~19 | 0 | 0 | . | . | . | . | . | . |
| 80~ vs 0~19 | 0 | 0 | . | . | . | . | . | . |
| **Gender** |  |  |  |  |  |  |  |  |
| F vs M | 0 | 0 | . | . | . | . | . | . |
| **Residence** |  |  |  |  |  |  |  |  |
| Kaohsiung | 0 | 0 | . | . | . | . | . | . |
| Tainan | 0 | 0 | . | . | . | . | . | . |
| Pingtung | 0 | 0 | . | . | . | . | . | . |
| New Taipei | 0 | 0 | . | . | . | . | . | . |
| Taipei | 0 | 0 | . | . | . | . | . | . |
| Taichung | 0 | 0 | . | . | . | . | . | . |
| Taoyuan | 0 | 0 | . | . | . | . | . | . |
| Penghu | 0 | 0 | . | . | . | . | . | . |
| Changhua | 0 | 0 | . | . | . | . | . | . |
| Yunlin | 0 | 0 | . | . | . | . | . | . |
| Hsinchu City | 0 | 0 | . | . | . | . | . | . |
| Nantou | 0 | 0 | . | . | . | . | . | . |
| Chiayi County | 0 | 0 | . | . | . | . | . | . |
| Miaoli | 0 | 0 | . | . | . | . | . | . |
| Taitung | 0 | 0 | . | . | . | . | . | . |
| Yilan | 0 | 0 | . | . | . | . | . | . |
| Keelung | 0 | 0 | . | . | . | . | . | . |
| Hsinchu City | 0 | 0 | . | . | . | . | . | . |
| Chiayi City | 0 | 0 | . | . | . | . | . | . |
| Hualien | 0 | 0 | . | . | . | . | . | . |
| Kinmen, Lienchiang | 0 | 0 | . | . | . | . | . | . |

Outcome: Sjögren’s syndrome

| Parameter | DF | Parameter Estimates | Standard Errors | Chi- Square | Pr>  ChiSq | Hazard Ratio | 95% Hazard Ratio Confidence Limits | |
| --- | --- | --- | --- | --- | --- | --- | --- | --- |
| **Group** |  |  |  |  |  |  |  |  |
| Dengue vs Comparison | 1 | -1.22 | 0.42 | 8.39 | 0.00 | 0.30 | 0.13 | 0.67 |
| **CCI score** |  |  |  |  |  |  |  |  |
| 1 vs 0 | 1 | 0.27 | 0.35 | 0.59 | 0.44 | 1.31 | 0.66 | 2.63 |
| 2~3 vs 0 | 1 | 1.02 | 0.38 | 7.28 | 0.01 | 2.78 | 1.32 | 5.85 |
| 4~6 vs 0 | 1 | 2.27 | 0.87 | 6.86 | 0.01 | 9.72 | 1.77 | 53.28 |
| 7~10 vs 0 | 1 | 2.99 | 1.04 | 8.26 | 0.00 | 19.98 | 2.59 | 154.02 |
| 11~ vs 0 | 1 | -14.22 | 2646 | 0 | 1.00 | 0 | 0 | . |
| **Periodontitis** |  |  |  |  |  |  |  |  |
| Yes vs No | 1 | 0.44 | 0.29 | 2.34 | 0.13 | 1.55 | 0.88 | 2.70 |
| **Age** |  |  |  |  |  |  |  |  |
| 20~29 vs 0~19 | 0 | 0 | . | . | . | . | . | . |
| 30~39 vs 0~19 | 1 | -0.17 | 2657 | 0 | 1.00 | 0.84 | 0 | . |
| 40~49 vs 0~19 | 1 | 0.46 | 2657 | 0 | 1.00 | 1.59 | 0 | . |
| 50~59 vs 0~19 | 1 | -0.27 | 2657 | 0 | 1.00 | 0.77 | 0 | . |
| 60~69 vs 0~19 | 1 | 15.04 | 2031 | 0.0001 | 0.99 | 3407928 | 0 | . |
| 70~79 vs 0~19 | 0 | 0 | . | . | . | . | . | . |
| 80~ vs 0~19 | 0 | 0 | . | . | . | . | . | . |
| **Gender** |  |  |  |  |  |  |  |  |
| F vs M | 0 | 0 | . | . | . | . | . | . |
| **Residence** |  |  |  |  |  |  |  |  |
| Kaohsiung | 0 | 0 | . | . | . | . | . | . |
| Tainan | 0 | 0 | . | . | . | . | . | . |
| Pingtung | 0 | 0 | . | . | . | . | . | . |
| New Taipei | 0 | 0 | . | . | . | . | . | . |
| Taipei | 0 | 0 | . | . | . | . | . | . |
| Taichung | 0 | 0 | . | . | . | . | . | . |
| Taoyuan | 0 | 0 | . | . | . | . | . | . |
| Penghu | 0 | 0 | . | . | . | . | . | . |
| Changhua | 0 | 0 | . | . | . | . | . | . |
| Yunlin | 0 | 0 | . | . | . | . | . | . |
| Hsinchu City | 0 | 0 | . | . | . | . | . | . |
| Nantou | 0 | 0 | . | . | . | . | . | . |
| Chiayi County | 0 | 0 | . | . | . | . | . | . |
| Miaoli | 0 | 0 | . | . | . | . | . | . |
| Taitung | 0 | 0 | . | . | . | . | . | . |
| Yilan | 0 | 0 | . | . | . | . | . | . |
| Keelung | 0 | 0 | . | . | . | . | . | . |
| Hsinchu City | 0 | 0 | . | . | . | . | . | . |
| Chiayi City | 0 | 0 | . | . | . | . | . | . |
| Hualien | 0 | 0 | . | . | . | . | . | . |
| Kinmen, Lienchiang | 0 | 0 | . | . | . | . | . | . |

Outcome: Systemic lupus erythematosus

| Parameter | DF | Parameter Estimates | Standard Errors | Chi- Square | Pr>  ChiSq | Hazard Ratio | 95% Hazard Ratio Confidence Limits | |
| --- | --- | --- | --- | --- | --- | --- | --- | --- |
| **Group** |  |  |  |  |  |  |  |  |
| Dengue vs Comparison | 1 | 0.50 | 0.41 | 1.48 | 0.22 | 1.65 | 0.74 | 3.71 |
| **CCI score** |  |  |  |  |  |  |  |  |
| 1 vs 0 | 1 | 1.17 | 0.49 | 5.72 | 0.02 | 3.22 | 1.24 | 8.41 |
| 2~3 vs 0 | 1 | 0.78 | 1.06 | 0.54 | 0.46 | 2.17 | 0.27 | 17.36 |
| 4~6 vs 0 | 1 | 0.38 | 1.21 | 0.10 | 0.75 | 1.46 | 0.14 | 15.53 |
| 7~10 vs 0 | 1 | -16.01 | 5218 | 0 | 0.998 | 0 | 0 | . |
| 11~ vs 0 | 0 | 0 | . | . | . | . | . | . |
| **Periodontitis** |  |  |  |  |  |  |  |  |
| Yes vs No | 1 | -1.69 | 0.43 | 15.23 | <.0001 | 0.19 | 0.08 | 0.43 |
| **Age** |  |  |  |  |  |  |  |  |
| 20~29 vs 0~19 | 1 | -16.01 | 5218 | 0 | 1.00 | 0 | 0 | . |
| 30~39 vs 0~19 | 1 | 15.98 | 3313 | 0 | 1.00 | 8723768 | 0 | . |
| 40~49 vs 0~19 | 0 | 0 | . | . | . | . | . | . |
| 50~59 vs 0~19 | 0 | 0 | . | . | . | . | . | . |
| 60~69 vs 0~19 | 1 | 18.11 | 2859 | 0 | 0.99 | 73305882 | 0 | . |
| 70~79 vs 0~19 | 0 | 0 | . | . | . | . | . | . |
| 80~ vs 0~19 | 0 | 0 | . | . | . | . | . | . |
| **Gender** |  |  |  |  |  |  |  |  |
| F vs M | 0 | 0 | . | . | . | . | . | . |
| **Residence** |  |  |  |  |  |  |  |  |
| Kaohsiung | 0 | 0 | . | . | . | . | . | . |
| Tainan | 0 | 0 | . | . | . | . | . | . |
| Pingtung | 0 | 0 | . | . | . | . | . | . |
| New Taipei | 0 | 0 | . | . | . | . | . | . |
| Taipei | 0 | 0 | . | . | . | . | . | . |
| Taichung | 0 | 0 | . | . | . | . | . | . |
| Taoyuan | 0 | 0 | . | . | . | . | . | . |
| Penghu | 0 | 0 | . | . | . | . | . | . |
| Changhua | 0 | 0 | . | . | . | . | . | . |
| Yunlin | 0 | 0 | . | . | . | . | . | . |
| Hsinchu City | 0 | 0 | . | . | . | . | . | . |
| Nantou | 0 | 0 | . | . | . | . | . | . |
| Chiayi County | 0 | 0 | . | . | . | . | . | . |
| Miaoli | 0 | 0 | . | . | . | . | . | . |
| Taitung | 0 | 0 | . | . | . | . | . | . |
| Yilan | 0 | 0 | . | . | . | . | . | . |
| Keelung | 0 | 0 | . | . | . | . | . | . |
| Hsinchu City | 0 | 0 | . | . | . | . | . | . |
| Chiayi City | 0 | 0 | . | . | . | . | . | . |
| Hualien | 0 | 0 | . | . | . | . | . | . |
| Kinmen, Lienchiang | 0 | 0 | . | . | . | . | . | . |

Outcome: Organ-specific autoimmune diseases

| Parameter | DF | Parameter Estimates | Standard Errors | Chi- Square | Pr>  ChiSq | Hazard Ratio | 95% Hazard Ratio Confidence Limits | |
| --- | --- | --- | --- | --- | --- | --- | --- | --- |
| **Group** |  |  |  |  |  |  |  |  |
| Dengue vs Comparison | 1 | 0.03 | 0.05 | 0.41 | 0.52 | 1.03 | 0.94 | 1.13 |
| **CCI score** |  |  |  |  |  |  |  |  |
| 1 vs 0 | 0.33 | 0.05 | 36.81 | <.0001 | 1.39 | 1.25 | 1.55 | 0.33 |
| 2~3 vs 0 | 0.69 | 0.07 | 95.78 | <.0001 | 2.00 | 1.74 | 2.30 | 0.69 |
| 4~6 vs 0 | 0.61 | 0.13 | 22.74 | <.0001 | 1.84 | 1.43 | 2.37 | 0.61 |
| 7~10 vs 0 | 0.69 | 0.26 | 6.98 | 0.0083 | 2.00 | 1.20 | 3.34 | 0.69 |
| 11~ vs 0 | 1.60 | 0.47 | 11.62 | 0.0007 | 4.94 | 1.97 | 12.39 | 1.60 |
| **Periodontitis** |  |  |  |  |  |  |  |  |
| Yes vs No | 1 | -0.47 | 0.04 | 110.56 | <.0001 | 0.63 | 0.58 | 0.69 |
| **Age** |  |  |  |  |  |  |  |  |
| 20~29 vs 0~19 | 1 | 1.85 | 1.19 | 2.44 | 0.12 | 6.39 | 0.62 | 65.61 |
| 30~39 vs 0~19 | 1 | 1.48 | 1.36 | 1.17 | 0.28 | 4.38 | 0.30 | 63.27 |
| 40~49 vs 0~19 | 1 | 1.23 | 1.46 | 0.71 | 0.40 | 3.43 | 0.20 | 60.56 |
| 50~59 vs 0~19 | 1 | 0.99 | 1.50 | 0.44 | 0.51 | 2.70 | 0.14 | 51.40 |
| 60~69 vs 0~19 | 1 | 0.79 | 1.53 | 0.26 | 0.61 | 2.20 | 0.11 | 44.51 |
| 70~79 vs 0~19 | 1 | 1.55 | 1.58 | 0.96 | 0.33 | 4.69 | 0.21 | 103.98 |
| 80~ vs 0~19 | 1 | -0.28 | 1.78 | 0.03 | 0.87 | 0.75 | 0.02 | 24.61 |
| **Gender** |  |  |  |  |  |  |  |  |
| F vs M | 0 | 0 | . | . | . | . | . | . |
| **Residence** |  |  |  |  |  |  |  |  |
| Kaohsiung | 0 | 0 | . | . | . | . | . | . |
| Tainan | 0 | 0 | . | . | . | . | . | . |
| Pingtung | 0 | 0 | . | . | . | . | . | . |
| New Taipei | 0 | 0 | . | . | . | . | . | . |
| Taipei | 0 | 0 | . | . | . | . | . | . |
| Taichung | 0 | 0 | . | . | . | . | . | . |
| Taoyuan | 0 | 0 | . | . | . | . | . | . |
| Penghu | 0 | 0 | . | . | . | . | . | . |
| Changhua | 0 | 0 | . | . | . | . | . | . |
| Yunlin | 0 | 0 | . | . | . | . | . | . |
| Hsinchu City | 0 | 0 | . | . | . | . | . | . |
| Nantou | 0 | 0 | . | . | . | . | . | . |
| Chiayi County | 0 | 0 | . | . | . | . | . | . |
| Miaoli | 0 | 0 | . | . | . | . | . | . |
| Taitung | 0 | 0 | . | . | . | . | . | . |
| Yilan | 0 | 0 | . | . | . | . | . | . |
| Keelung | 0 | 0 | . | . | . | . | . | . |
| Hsinchu City | 0 | 0 | . | . | . | . | . | . |
| Chiayi City | 0 | 0 | . | . | . | . | . | . |
| Hualien | 0 | 0 | . | . | . | . | . | . |
| Kinmen, Lienchiang | 0 | 0 | . | . | . | . | . | . |

Outcome: Addison’s disease

| Parameter | DF | Parameter Estimates | Standard Errors | Chi- Square | Pr>  ChiSq | Hazard Ratio | 95% Hazard Ratio Confidence Limits | |
| --- | --- | --- | --- | --- | --- | --- | --- | --- |
| **Group** |  |  |  |  |  |  |  |  |
| Dengue vs Comparison | 1 | -0.04 | 0.14 | 0.09 | 0.76 | 0.96 | 0.74 | 1.25 |
| **CCI score** |  |  |  |  |  |  |  |  |
| 1 vs 0 | 1 | 0.54 | 0.14 | 13.94 | 0.0002 | 1.72 | 1.29 | 2.28 |
| 2~3 vs 0 | 1 | 0.89 | 0.18 | 25.46 | <.0001 | 2.44 | 1.73 | 3.45 |
| 4~6 vs 0 | 1 | 0.91 | 0.31 | 8.88 | 0.0029 | 2.49 | 1.37 | 4.52 |
| 7~10 vs 0 | 1 | 1.63 | 0.52 | 9.73 | 0.0018 | 5.11 | 1.83 | 14.24 |
| 11~ vs 0 | 1 | 1.37 | 0.74 | 3.44 | 0.0634 | 3.92 | 0.93 | 16.58 |
| **Periodontitis** |  |  |  |  |  |  |  |  |
| Yes vs No | 1 | -0.77 | 0.12 | 39.31 | <.0001 | 0.46 | 0.36 | 0.59 |
| **Age** |  |  |  |  |  |  |  |  |
| 20~29 vs 0~19 | 0 | 0 | . | . | . | . | . | . |
| 30~39 vs 0~19 | 0 | 0 | . | . | . | . | . | . |
| 40~49 vs 0~19 | 1 | 1.73 | 2.20 | 0.62 | 0.43 | 5.62 | 0.08 | 419.75 |
| 50~59 vs 0~19 | 1 | 0.20 | 1.76 | 0.01 | 0.91 | 1.23 | 0.04 | 38.52 |
| 60~69 vs 0~19 | 1 | 0.39 | 1.52 | 0.07 | 0.80 | 1.48 | 0.08 | 29.19 |
| 70~79 vs 0~19 | 1 | 0.83 | 1.35 | 0.38 | 0.54 | 2.29 | 0.16 | 32.22 |
| 80~ vs 0~19 | 0 | 0 | . | . | . | . | . | . |
| **Gender** |  |  |  |  |  |  |  |  |
| F vs M | 0 | 0 | . | . | . | . | . | . |
| **Residence** |  |  |  |  |  |  |  |  |
| Kaohsiung | 0 | 0 | . | . | . | . | . | . |
| Tainan | 0 | 0 | . | . | . | . | . | . |
| Pingtung | 0 | 0 | . | . | . | . | . | . |
| New Taipei | 0 | 0 | . | . | . | . | . | . |
| Taipei | 0 | 0 | . | . | . | . | . | . |
| Taichung | 0 | 0 | . | . | . | . | . | . |
| Taoyuan | 0 | 0 | . | . | . | . | . | . |
| Penghu | 0 | 0 | . | . | . | . | . | . |
| Changhua | 0 | 0 | . | . | . | . | . | . |
| Yunlin | 0 | 0 | . | . | . | . | . | . |
| Hsinchu City | 0 | 0 | . | . | . | . | . | . |
| Nantou | 0 | 0 | . | . | . | . | . | . |
| Chiayi County | 0 | 0 | . | . | . | . | . | . |
| Miaoli | 0 | 0 | . | . | . | . | . | . |
| Taitung | 0 | 0 | . | . | . | . | . | . |
| Yilan | 0 | 0 | . | . | . | . | . | . |
| Keelung | 0 | 0 | . | . | . | . | . | . |
| Hsinchu City | 0 | 0 | . | . | . | . | . | . |
| Chiayi City | 0 | 0 | . | . | . | . | . | . |
| Hualien | 0 | 0 | . | . | . | . | . | . |
| Kinmen, Lienchiang | 0 | 0 | . | . | . | . | . | . |

Outcome: Autoimmune hemolytic anemia

| Parameter | DF | Parameter Estimates | Standard Errors | Chi- Square | Pr>  ChiSq | Hazard Ratio | 95% Hazard Ratio Confidence Limits | |
| --- | --- | --- | --- | --- | --- | --- | --- | --- |
| **Group** |  |  |  |  |  |  |  |  |
| Dengue vs Comparison | 1 | 0.07 | 0.60 | 0.01 | 0.91 | 1.07 | 0.33 | 3.47 |
| **CCI score** |  |  |  |  |  |  |  |  |
| 1 vs 0 | 1 | -0.81 | 0.69 | 1.37 | 0.24 | 0.45 | 0.11 | 1.73 |
| 2~3 vs 0 | 1 | -0.30 | 1.23 | 0.06 | 0.81 | 0.74 | 0.07 | 8.23 |
| 4~6 vs 0 | 1 | -16.83 | 3314 | 0 | 0.996 | 0 | 0 | . |
| 7~10 vs 0 | 0 | 0 | . | . | . | . | . | . |
| 11~ vs 0 | 1 | 19.87 | 11026 | 0 | 0.999 | 4.27E+08 | 0 | . |
| **Periodontitis** |  |  |  |  |  |  |  |  |
| Yes vs No | 1 | -1.27 | 0.68 | 3.45 | 0.06 | 0.28 | 0.07 | 1.07 |
| **Age** |  |  |  |  |  |  |  |  |
| 20~29 vs 0~19 | 0 | 0 | . | . | . | . | . | . |
| 30~39 vs 0~19 | 0 | 0 | . | . | . | . | . | . |
| 40~49 vs 0~19 | 0 | 0 | . | . | . | . | . | . |
| 50~59 vs 0~19 | 0 | 0 | . | . | . | . | . | . |
| 60~69 vs 0~19 | 0 | 0 | . | . | . | . | . | . |
| 70~79 vs 0~19 | 0 | 0 | . | . | . | . | . | . |
| 80~ vs 0~19 | 0 | 0 | . | . | . | . | . | . |
| **Gender** |  |  |  |  |  |  |  |  |
| F vs M | 0 | 0 | . | . | . | . | . | . |
| **Residence** |  |  |  |  |  |  |  |  |
| Kaohsiung | 0 | 0 | . | . | . | . | . | . |
| Tainan | 0 | 0 | . | . | . | . | . | . |
| Pingtung | 0 | 0 | . | . | . | . | . | . |
| New Taipei | 0 | 0 | . | . | . | . | . | . |
| Taipei | 0 | 0 | . | . | . | . | . | . |
| Taichung | 0 | 0 | . | . | . | . | . | . |
| Taoyuan | 0 | 0 | . | . | . | . | . | . |
| Penghu | 0 | 0 | . | . | . | . | . | . |
| Changhua | 0 | 0 | . | . | . | . | . | . |
| Yunlin | 0 | 0 | . | . | . | . | . | . |
| Hsinchu City | 0 | 0 | . | . | . | . | . | . |
| Nantou | 0 | 0 | . | . | . | . | . | . |
| Chiayi County | 0 | 0 | . | . | . | . | . | . |
| Miaoli | 0 | 0 | . | . | . | . | . | . |
| Taitung | 0 | 0 | . | . | . | . | . | . |
| Yilan | 0 | 0 | . | . | . | . | . | . |
| Keelung | 0 | 0 | . | . | . | . | . | . |
| Hsinchu City | 0 | 0 | . | . | . | . | . | . |
| Chiayi City | 0 | 0 | . | . | . | . | . | . |
| Hualien | 0 | 0 | . | . | . | . | . | . |
| Kinmen, Lienchiang | 0 | 0 | . | . | . | . | . | . |

Outcome: Diabetes mellitus type 1

| Parameter | DF | Parameter Estimates | Standard Errors | Chi- Square | Pr>  ChiSq | Hazard Ratio | 95% Hazard Ratio Confidence Limits | |
| --- | --- | --- | --- | --- | --- | --- | --- | --- |
| **Group** |  |  |  |  |  |  |  |  |
| Dengue vs Comparison | 1 | 0.00019 | 0.08 | 0 | 0.998 | 1.00 | 0.86 | 1.16 |
| **CCI score** |  |  |  |  |  |  |  |  |
| 1 vs 0 | 1 | 0.37 | 0.08 | 19.03 | <.0001 | 1.45 | 1.23 | 1.71 |
| 2~3 vs 0 | 1 | 0.77 | 0.11 | 49.90 | <.0001 | 2.16 | 1.75 | 2.68 |
| 4~6 vs 0 | 1 | 0.30 | 0.21 | 2.14 | 0.14 | 1.35 | 0.90 | 2.02 |
| 7~10 vs 0 | 1 | 0.66 | 0.45 | 2.15 | 0.14 | 1.93 | 0.80 | 4.65 |
| 11~ vs 0 | 1 | 1.43 | 0.68 | 4.42 | 0.04 | 4.18 | 1.10 | 15.87 |
| **Periodontitis** |  |  |  |  |  |  |  |  |
| Yes vs No | 1 | -0.56 | 0.07 | 63.58 | <.0001 | 0.57 | 0.50 | 0.66 |
| **Age** |  |  |  |  |  |  |  |  |
| 20~29 vs 0~19 | 1 | 14.78 | 349.54 | 0.0018 | 0.97 | 2618524.0 | 0 | 8.89E+303 |
| 30~39 vs 0~19 | 1 | 14.38 | 349.54 | 0.0017 | 0.97 | 1752758.0 | 0 | 5.92E+303 |
| 40~49 vs 0~19 | 1 | 13.42 | 349.54 | 0.0015 | 0.97 | 671673.5 | 0 | 2.26E+303 |
| 50~59 vs 0~19 | 1 | 13.07 | 349.54 | 0.0014 | 0.97 | 473884.1 | 0 | 1.59E+303 |
| 60~69 vs 0~19 | 1 | 12.69 | 349.54 | 0.0013 | 0.97 | 324296.6 | 0 | 1.09E+303 |
| 70~79 vs 0~19 | 1 | 13.45 | 349.54 | 0.0015 | 0.97 | 695961 | 0 | 2.34E+303 |
| 80~ vs 0~19 | 0 | 0 | . | . | . | . | . | . |
| **Gender** |  |  |  |  |  |  |  |  |
| F vs M | 0 | 0 | . | . | . | . | . | . |
| **Residence** |  |  |  |  |  |  |  |  |
| Kaohsiung | 0 | 0 | . | . | . | . | . | . |
| Tainan | 0 | 0 | . | . | . | . | . | . |
| Pingtung | 0 | 0 | . | . | . | . | . | . |
| New Taipei | 0 | 0 | . | . | . | . | . | . |
| Taipei | 0 | 0 | . | . | . | . | . | . |
| Taichung | 0 | 0 | . | . | . | . | . | . |
| Taoyuan | 0 | 0 | . | . | . | . | . | . |
| Penghu | 0 | 0 | . | . | . | . | . | . |
| Changhua | 0 | 0 | . | . | . | . | . | . |
| Yunlin | 0 | 0 | . | . | . | . | . | . |
| Hsinchu City | 0 | 0 | . | . | . | . | . | . |
| Nantou | 0 | 0 | . | . | . | . | . | . |
| Chiayi County | 0 | 0 | . | . | . | . | . | . |
| Miaoli | 0 | 0 | . | . | . | . | . | . |
| Taitung | 0 | 0 | . | . | . | . | . | . |
| Yilan | 0 | 0 | . | . | . | . | . | . |
| Keelung | 0 | 0 | . | . | . | . | . | . |
| Hsinchu City | 0 | 0 | . | . | . | . | . | . |
| Chiayi City | 0 | 0 | . | . | . | . | . | . |
| Hualien | 0 | 0 | . | . | . | . | . | . |
| Kinmen, Lienchiang | 0 | 0 | . | . | . | . | . | . |

Outcome: Graves’ disease

| Parameter | DF | Parameter Estimates | Standard Errors | Chi- Square | Pr>  ChiSq | Hazard Ratio | 95% Hazard Ratio Confidence Limits | |
| --- | --- | --- | --- | --- | --- | --- | --- | --- |
| **Group** |  |  |  |  |  |  |  |  |
| Dengue vs Comparison | 1 | 0.11 | 0.13 | 0.75 | 0.39 | 1.12 | 0.87 | 1.45 |
| **CCI score** |  |  |  |  |  |  |  |  |
| 1 vs 0 | 1 | 0.19 | 0.16 | 1.35 | 0.25 | 1.21 | 0.88 | 1.65 |
| 2~3 vs 0 | 1 | 0.38 | 0.23 | 2.65 | 0.10 | 1.46 | 0.93 | 2.29 |
| 4~6 vs 0 | 1 | -0.28 | 0.55 | 0.25 | 0.61 | 0.76 | 0.26 | 2.24 |
| 7~10 vs 0 | 1 | -13.32 | 665.91 | 0.0004 | 0.98 | 0 | 0 | . |
| 11~ vs 0 | 0 | 0 | . | . | . | . | . | . |
| **Periodontitis** |  |  |  |  |  |  |  |  |
| Yes vs No | 1 | -0.52 | 0.12 | 17.41 | <.0001 | 0.59 | 0.47 | 0.76 |
| **Age** |  |  |  |  |  |  |  |  |
| 20~29 vs 0~19 | 1 | 1.20 | 1.44 | 0.69 | 0.41 | 3.31 | 0.20 | 55.83 |
| 30~39 vs 0~19 | 1 | 15.44 | 865.91 | 0.0003 | 0.99 | 5096745 | 0 | . |
| 40~49 vs 0~19 | 1 | 16.75 | 865.91 | 0.0004 | 0.98 | 18752667 | 0 | . |
| 50~59 vs 0~19 | 1 | 17.25 | 865.91 | 0.0004 | 0.98 | 30902829 | 0 | . |
| 60~69 vs 0~19 | 1 | 30.93 | 1717 | 0.0003 | 0.99 | 2.72E+13 | 0 | . |
| 70~79 vs 0~19 | 1 | 13.59 | 1265 | 0.0001 | 0.99 | 797070.2 | 0 | . |
| 80~ vs 0~19 | 0 | 0 | . | . | . | . | . | . |
| **Gender** |  |  |  |  |  |  |  |  |
| F vs M | 0 | 0 | . | . | . | . | . | . |
| **Residence** |  |  |  |  |  |  |  |  |
| Kaohsiung | 0 | 0 | . | . | . | . | . | . |
| Tainan | 0 | 0 | . | . | . | . | . | . |
| Pingtung | 0 | 0 | . | . | . | . | . | . |
| New Taipei | 0 | 0 | . | . | . | . | . | . |
| Taipei | 0 | 0 | . | . | . | . | . | . |
| Taichung | 0 | 0 | . | . | . | . | . | . |
| Taoyuan | 0 | 0 | . | . | . | . | . | . |
| Penghu | 0 | 0 | . | . | . | . | . | . |
| Changhua | 0 | 0 | . | . | . | . | . | . |
| Yunlin | 0 | 0 | . | . | . | . | . | . |
| Hsinchu City | 0 | 0 | . | . | . | . | . | . |
| Nantou | 0 | 0 | . | . | . | . | . | . |
| Chiayi County | 0 | 0 | . | . | . | . | . | . |
| Miaoli | 0 | 0 | . | . | . | . | . | . |
| Taitung | 0 | 0 | . | . | . | . | . | . |
| Yilan | 0 | 0 | . | . | . | . | . | . |
| Keelung | 0 | 0 | . | . | . | . | . | . |
| Hsinchu City | 0 | 0 | . | . | . | . | . | . |
| Chiayi City | 0 | 0 | . | . | . | . | . | . |
| Hualien | 0 | 0 | . | . | . | . | . | . |
| Kinmen, Lienchiang | 0 | 0 | . | . | . | . | . | . |

Outcome: Hashimoto’ss thyroiditis

| Parameter | DF | Parameter Estimates | Standard Errors | Chi- Square | Pr>  ChiSq | Hazard Ratio | 95% Hazard Ratio Confidence Limits | |
| --- | --- | --- | --- | --- | --- | --- | --- | --- |
| **Group** |  |  |  |  |  |  |  |  |
| Dengue vs Comparison | 1 | -0.11 | 0.11 | 0.9241 | 0.34 | 0.90 | 0.72 | 1.12 |
| **CCI score** |  |  |  |  |  |  |  |  |
| 1 vs 0 | 1 | 0.36 | 0.13 | 7.80 | 0.0052 | 1.43 | 1.11 | 1.85 |
| 2~3 vs 0 | 1 | 0.73 | 0.16 | 21.38 | <.0001 | 2.07 | 1.52 | 2.83 |
| 4~6 vs 0 | 1 | 1.28 | 0.28 | 20.59 | <.0001 | 3.61 | 2.08 | 6.29 |
| 7~10 vs 0 | 1 | 1.15 | 0.53 | 4.68 | 0.03 | 3.16 | 1.11 | 8.95 |
| 11~ vs 0 | 1 | 2.21 | 1.23 | 3.20 | 0.07 | 9.08 | 0.81 | 101.89 |
| **Periodontitis** |  |  |  |  |  |  |  |  |
| Yes vs No | 1 | -0.09 | 0.10 | 0.85 | 0.36 | 0.91 | 0.75 | 1.11 |
| **Age** |  |  |  |  |  |  |  |  |
| 20~29 vs 0~19 | 1 | -9.88 | 355.52 | 0.0008 | 0.98 | 0 | 0 | 2.14E+298 |
| 30~39 vs 0~19 | 1 | 0.46 | 2.32 | 0.0393 | 0.84 | 1.59 | 0.02 | 150.738 |
| 40~49 vs 0~19 | 1 | -0.40 | 2.08 | 0.0361 | 0.85 | 0.67 | 0.01 | 39.854 |
| 50~59 vs 0~19 | 1 | -0.46 | 1.95 | 0.0547 | 0.82 | 0.63 | 0.01 | 28.888 |
| 60~69 vs 0~19 | 1 | 0.06 | 1.85 | 0.0009 | 0.98 | 1.06 | 0.03 | 39.531 |
| 70~79 vs 0~19 | 1 | -0.14 | 1.34 | 0.0102 | 0.92 | 0.87 | 0.06 | 12.018 |
| 80~ vs 0~19 | 0 | 0 | . | . | . | . | . | . |
| **Gender** |  |  |  |  |  |  |  |  |
| F vs M | 0 | 0 | . | . | . | . | . | . |
| **Residence** |  |  |  |  |  |  |  |  |
| Kaohsiung | 0 | 0 | . | . | . | . | . | . |
| Tainan | 0 | 0 | . | . | . | . | . | . |
| Pingtung | 0 | 0 | . | . | . | . | . | . |
| New Taipei | 0 | 0 | . | . | . | . | . | . |
| Taipei | 0 | 0 | . | . | . | . | . | . |
| Taichung | 0 | 0 | . | . | . | . | . | . |
| Taoyuan | 0 | 0 | . | . | . | . | . | . |
| Penghu | 0 | 0 | . | . | . | . | . | . |
| Changhua | 0 | 0 | . | . | . | . | . | . |
| Yunlin | 0 | 0 | . | . | . | . | . | . |
| Hsinchu City | 0 | 0 | . | . | . | . | . | . |
| Nantou | 0 | 0 | . | . | . | . | . | . |
| Chiayi County | 0 | 0 | . | . | . | . | . | . |
| Miaoli | 0 | 0 | . | . | . | . | . | . |
| Taitung | 0 | 0 | . | . | . | . | . | . |
| Yilan | 0 | 0 | . | . | . | . | . | . |
| Keelung | 0 | 0 | . | . | . | . | . | . |
| Hsinchu City | 0 | 0 | . | . | . | . | . | . |
| Chiayi City | 0 | 0 | . | . | . | . | . | . |
| Hualien | 0 | 0 | . | . | . | . | . | . |
| Kinmen, Lienchiang | 0 | 0 | . | . | . | . | . | . |

Outcome: Henoch–Schonlein purpura

| Parameter | DF | Parameter Estimates | Standard Errors | Chi- Square | Pr>  ChiSq | Hazard Ratio | 95% Hazard Ratio Confidence Limits | |
| --- | --- | --- | --- | --- | --- | --- | --- | --- |
| **Group** |  |  |  |  |  |  |  |  |
| Dengue vs Comparison | 1 | 0.79 | 0.50 | 2.53 | 0.11 | 2.20 | 0.83 | 5.81 |
| **CCI score** |  |  |  |  |  |  |  |  |
| 1 vs 0 | 1 | 0.45 | 0.59 | 0.59 | 0.44 | 1.57 | 0.50 | 4.95 |
| 2~3 vs 0 | 1 | 0.99 | 1.05 | 0.89 | 0.34 | 2.70 | 0.35 | 21.11 |
| 4~6 vs 0 | 0 | 0 | . | . | . | . | . | . |
| 7~10 vs 0 | 0 | 0 | . | . | . | . | . | . |
| 11~ vs 0 | 0 | 0 | . | . | . | . | . | . |
| **Periodontitis** |  |  |  |  |  |  |  |  |
| Yes vs No | 1 | 1.22 | 0.74 | 2.73 | 0.10 | 3.38 | 0.80 | 14.34 |
| **Age** |  |  |  |  |  |  |  |  |
| 20~29 vs 0~19 | 0 | 0 | . | . | . | . | . | . |
| 30~39 vs 0~19 | 0 | 0 | . | . | . | . | . | . |
| 40~49 vs 0~19 | 0 | 0 | . | . | . | . | . | . |
| 50~59 vs 0~19 | 1 | -14.53 | 2425 | 0 | 1.00 | 0 | 0 | . |
| 60~69 vs 0~19 | 0 | 0 | . | . | . | . | . | . |
| 70~79 vs 0~19 | 0 | 0 | . | . | . | . | . | . |
| 80~ vs 0~19 | 0 | 0 | . | . | . | . | . | . |
| **Gender** |  |  |  |  |  |  |  |  |
| F vs M | 0 | 0 | . | . | . | . | . | . |
| **Residence** |  |  |  |  |  |  |  |  |
| Kaohsiung | 0 | 0 | . | . | . | . | . | . |
| Tainan | 0 | 0 | . | . | . | . | . | . |
| Pingtung | 0 | 0 | . | . | . | . | . | . |
| New Taipei | 0 | 0 | . | . | . | . | . | . |
| Taipei | 0 | 0 | . | . | . | . | . | . |
| Taichung | 0 | 0 | . | . | . | . | . | . |
| Taoyuan | 0 | 0 | . | . | . | . | . | . |
| Penghu | 0 | 0 | . | . | . | . | . | . |
| Changhua | 0 | 0 | . | . | . | . | . | . |
| Yunlin | 0 | 0 | . | . | . | . | . | . |
| Hsinchu City | 0 | 0 | . | . | . | . | . | . |
| Nantou | 0 | 0 | . | . | . | . | . | . |
| Chiayi County | 0 | 0 | . | . | . | . | . | . |
| Miaoli | 0 | 0 | . | . | . | . | . | . |
| Taitung | 0 | 0 | . | . | . | . | . | . |
| Yilan | 0 | 0 | . | . | . | . | . | . |
| Keelung | 0 | 0 | . | . | . | . | . | . |
| Hsinchu City | 0 | 0 | . | . | . | . | . | . |
| Chiayi City | 0 | 0 | . | . | . | . | . | . |
| Hualien | 0 | 0 | . | . | . | . | . | . |
| Kinmen, Lienchiang | 0 | 0 | . | . | . | . | . | . |

Outcome: Immune thrombocytopenic purpura

| Parameter | DF | Parameter Estimates | Standard Errors | Chi- Square | Pr>  ChiSq | Hazard Ratio | 95% Hazard Ratio Confidence Limits | |
| --- | --- | --- | --- | --- | --- | --- | --- | --- |
| **Group** |  |  |  |  |  |  |  |  |
| Dengue vs Comparison | 1 | 0.46 | 0.27 | 2.98 | 0.08 | 1.59 | 0.94 | 2.69 |
| **CCI score** |  |  |  |  |  |  |  |  |
| 1 vs 0 | 1 | -0.45 | 0.40 | 1.24 | 0.27 | 0.64 | 0.29 | 1.40 |
| 2~3 vs 0 | 1 | 0.60 | 0.43 | 1.96 | 0.16 | 1.83 | 0.79 | 4.25 |
| 4~6 vs 0 | 1 | 0.74 | 0.67 | 1.24 | 0.27 | 2.10 | 0.57 | 7.74 |
| 7~10 vs 0 | 0 | 0 | . | . | . | . | . | . |
| 11~ vs 0 | 0 | 0 | . | . | . | . | . | . |
| **Periodontitis** |  |  |  |  |  |  |  |  |
| Yes vs No | 1 | -0.46 | 0.29 | 2.59 | 0.11 | 0.63 | 0.36 | 1.11 |
| **Age** |  |  |  |  |  |  |  |  |
| 20~29 vs 0~19 | 1 | 16.64 | 3231 | 0 | 1.00 | 16862670 | 0 | . |
| 30~39 vs 0~19 | 1 | 0.84 | 4010 | 0 | 1.00 | 2.33 | 0 | . |
| 40~49 vs 0~19 | 1 | -17.01 | 5254 | 0 | 1.00 | 0 | 0 | . |
| 50~59 vs 0~19 | 1 | -0.30 | 4030 | 0 | 1.00 | 0.74 | 0 | . |
| 60~69 vs 0~19 | 1 | -15.81 | 3280 | 0 | 1.00 | 0 | 0 | . |
| 70~79 vs 0~19 | 0 | 0 | . | . | . | . | . | . |
| 80~ vs 0~19 | 0 | 0 | . | . | . | . | . | . |
| **Gender** |  |  |  |  |  |  |  |  |
| F vs M | 0 | 0 | . | . | . | . | . | . |
| **Residence** |  |  |  |  |  |  |  |  |
| Kaohsiung | 0 | 0 | . | . | . | . | . | . |
| Tainan | 0 | 0 | . | . | . | . | . | . |
| Pingtung | 0 | 0 | . | . | . | . | . | . |
| New Taipei | 0 | 0 | . | . | . | . | . | . |
| Taipei | 0 | 0 | . | . | . | . | . | . |
| Taichung | 0 | 0 | . | . | . | . | . | . |
| Taoyuan | 0 | 0 | . | . | . | . | . | . |
| Penghu | 0 | 0 | . | . | . | . | . | . |
| Changhua | 0 | 0 | . | . | . | . | . | . |
| Yunlin | 0 | 0 | . | . | . | . | . | . |
| Hsinchu City | 0 | 0 | . | . | . | . | . | . |
| Nantou | 0 | 0 | . | . | . | . | . | . |
| Chiayi County | 0 | 0 | . | . | . | . | . | . |
| Miaoli | 0 | 0 | . | . | . | . | . | . |
| Taitung | 0 | 0 | . | . | . | . | . | . |
| Yilan | 0 | 0 | . | . | . | . | . | . |
| Keelung | 0 | 0 | . | . | . | . | . | . |
| Hsinchu City | 0 | 0 | . | . | . | . | . | . |
| Chiayi City | 0 | 0 | . | . | . | . | . | . |
| Hualien | 0 | 0 | . | . | . | . | . | . |
| Kinmen, Lienchiang | 0 | 0 | . | . | . | . | . | . |

Outcome: Autoimmune hepatitis

| Parameter | DF | Parameter Estimates | Standard Errors | Chi- Square | Pr>  ChiSq | Hazard Ratio | 95% Hazard Ratio Confidence Limits | |
| --- | --- | --- | --- | --- | --- | --- | --- | --- |
| **Group** |  |  |  |  |  |  |  |  |
| Dengue vs Comparison | 1 | 0.20 | 0.12 | 2.51 | 0.11 | 1.22 | 0.95 | 1.56 |
| **CCI score** |  |  |  |  |  |  |  |  |
| 1 vs 0 | 1 | 0.25 | 0.15 | 2.85 | 0.09 | 1.29 | 0.96 | 1.72 |
| 2~3 vs 0 | 1 | 0.55 | 0.21 | 7.07 | 0.0078 | 1.73 | 1.16 | 2.58 |
| 4~6 vs 0 | 1 | 0.91 | 0.39 | 5.46 | 0.0194 | 2.47 | 1.16 | 5.29 |
| 7~10 vs 0 | 1 | -0.98 | 1.04 | 0.88 | 0.35 | 0.38 | 0.05 | 2.90 |
| 11~ vs 0 | 0 | 0 | . | . | . | . | . | . |
| **Periodontitis** |  |  |  |  |  |  |  |  |
| Yes vs No | 1 | -0.39 | 0.12 | 10.86 | 0.001 | 0.68 | 0.54 | 0.86 |
| **Age** |  |  |  |  |  |  |  |  |
| 20~29 vs 0~19 | 1 | 13.69 | 1136 | 0.0001 | 0.99 | 882855.50 | 0 | . |
| 30~39 vs 0~19 | 1 | 12.53 | 1136 | 0.0001 | 0.99 | 275755.60 | 0 | . |
| 40~49 vs 0~19 | 1 | 12.72 | 1136 | 0.0001 | 0.99 | 333412.20 | 0 | . |
| 50~59 vs 0~19 | 1 | 11.89 | 1136 | 0.0001 | 0.99 | 145418.30 | 0 | . |
| 60~69 vs 0~19 | 1 | 10.47 | 1136 | 0.0001 | 0.99 | 35373.57 | 0 | . |
| 70~79 vs 0~19 | 1 | 24.99 | 1428 | 0.0003 | 0.99 | 7.12E+10 | 0 | . |
| 80~ vs 0~19 | 1 | 7.68 | 2795 | 0 | 1.00 | 2162.52 | 0 | . |
| **Gender** |  |  |  |  |  |  |  |  |
| F vs M | 0 | 0 | . | . | . | . | . | . |
| **Residence** |  |  |  |  |  |  |  |  |
| Kaohsiung | 0 | 0 | . | . | . | . | . | . |
| Tainan | 0 | 0 | . | . | . | . | . | . |
| Pingtung | 0 | 0 | . | . | . | . | . | . |
| New Taipei | 0 | 0 | . | . | . | . | . | . |
| Taipei | 0 | 0 | . | . | . | . | . | . |
| Taichung | 0 | 0 | . | . | . | . | . | . |
| Taoyuan | 0 | 0 | . | . | . | . | . | . |
| Penghu | 0 | 0 | . | . | . | . | . | . |
| Changhua | 0 | 0 | . | . | . | . | . | . |
| Yunlin | 0 | 0 | . | . | . | . | . | . |
| Hsinchu City | 0 | 0 | . | . | . | . | . | . |
| Nantou | 0 | 0 | . | . | . | . | . | . |
| Chiayi County | 0 | 0 | . | . | . | . | . | . |
| Miaoli | 0 | 0 | . | . | . | . | . | . |
| Taitung | 0 | 0 | . | . | . | . | . | . |
| Yilan | 0 | 0 | . | . | . | . | . | . |
| Keelung | 0 | 0 | . | . | . | . | . | . |
| Hsinchu City | 0 | 0 | . | . | . | . | . | . |
| Chiayi City | 0 | 0 | . | . | . | . | . | . |
| Hualien | 0 | 0 | . | . | . | . | . | . |
| Kinmen, Lienchiang | 0 | 0 | . | . | . | . | . | . |

Outcome: Myasthenia gravis

| Parameter | DF | Parameter Estimates | Standard Errors | Chi- Square | Pr>  ChiSq | Hazard Ratio | 95% Hazard Ratio Confidence Limits | |
| --- | --- | --- | --- | --- | --- | --- | --- | --- |
| **Group** |  |  |  |  |  |  |  |  |
| Dengue vs Comparison | 1 | -0.17 | 0.67 | 0.06 | 0.80 | 0.85 | 0.23 | 3.14 |
| **CCI score** |  |  |  |  |  |  |  |  |
| 1 vs 0 | 1 | 0.42 | 0.70 | 0.37 | 0.54 | 1.53 | 0.39 | 5.98 |
| 2~3 vs 0 | 1 | -16.12 | 3235.00 | 0 | 1.00 | 0 | 0 | . |
| 4~6 vs 0 | 1 | 0.37 | 1.30 | 0.08 | 0.77 | 1.45 | 0.11 | 18.68 |
| 7~10 vs 0 | 0 | 0 | . | . | . | . | . | . |
| 11~ vs 0 | 0 | 0 | . | . | . | . | . | . |
| **Periodontitis** |  |  |  |  |  |  |  |  |
| Yes vs No | 1 | 0.34 | 0.63 | 0.30 | 0.58 | 1.41 | 0.41 | 4.81 |
| **Age** |  |  |  |  |  |  |  |  |
| 20~29 vs 0~19 | 0 | 0 | . | . | . | . | . | . |
| 30~39 vs 0~19 | 0 | 0 | . | . | . | . | . | . |
| 40~49 vs 0~19 | 0 | 0 | . | . | . | . | . | . |
| 50~59 vs 0~19 | 1 | 16.19 | 7196 | 0 | 1.00 | 10742138 | 0 | . |
| 60~69 vs 0~19 | 0 | 0 | . | . | . | . | . | . |
| 70~79 vs 0~19 | 0 | 0 | . | . | . | . | . | . |
| 80~ vs 0~19 | 0 | 0 | . | . | . | . | . | . |
| **Gender** |  |  |  |  |  |  |  |  |
| F vs M | 0 | 0 | . | . | . | . | . | . |
| **Residence** |  |  |  |  |  |  |  |  |
| Kaohsiung | 0 | 0 | . | . | . | . | . | . |
| Tainan | 0 | 0 | . | . | . | . | . | . |
| Pingtung | 0 | 0 | . | . | . | . | . | . |
| New Taipei | 0 | 0 | . | . | . | . | . | . |
| Taipei | 0 | 0 | . | . | . | . | . | . |
| Taichung | 0 | 0 | . | . | . | . | . | . |
| Taoyuan | 0 | 0 | . | . | . | . | . | . |
| Penghu | 0 | 0 | . | . | . | . | . | . |
| Changhua | 0 | 0 | . | . | . | . | . | . |
| Yunlin | 0 | 0 | . | . | . | . | . | . |
| Hsinchu City | 0 | 0 | . | . | . | . | . | . |
| Nantou | 0 | 0 | . | . | . | . | . | . |
| Chiayi County | 0 | 0 | . | . | . | . | . | . |
| Miaoli | 0 | 0 | . | . | . | . | . | . |
| Taitung | 0 | 0 | . | . | . | . | . | . |
| Yilan | 0 | 0 | . | . | . | . | . | . |
| Keelung | 0 | 0 | . | . | . | . | . | . |
| Hsinchu City | 0 | 0 | . | . | . | . | . | . |
| Chiayi City | 0 | 0 | . | . | . | . | . | . |
| Hualien | 0 | 0 | . | . | . | . | . | . |
| Kinmen, Lienchiang | 0 | 0 | . | . | . | . | . | . |
